# Supplementary figures and images for: Evaluation of the association of physical activity levels with self-perceived health, depression, and anxiety in Spanish individuals with high cholesterol levels: a retrospective cross-sectional study
Source: PeerJ. 2024 Jul 15;12:e17169. doi: 10.7717/peerj.17169 (PMC11257045; doi:10.7717/peerj.17169)

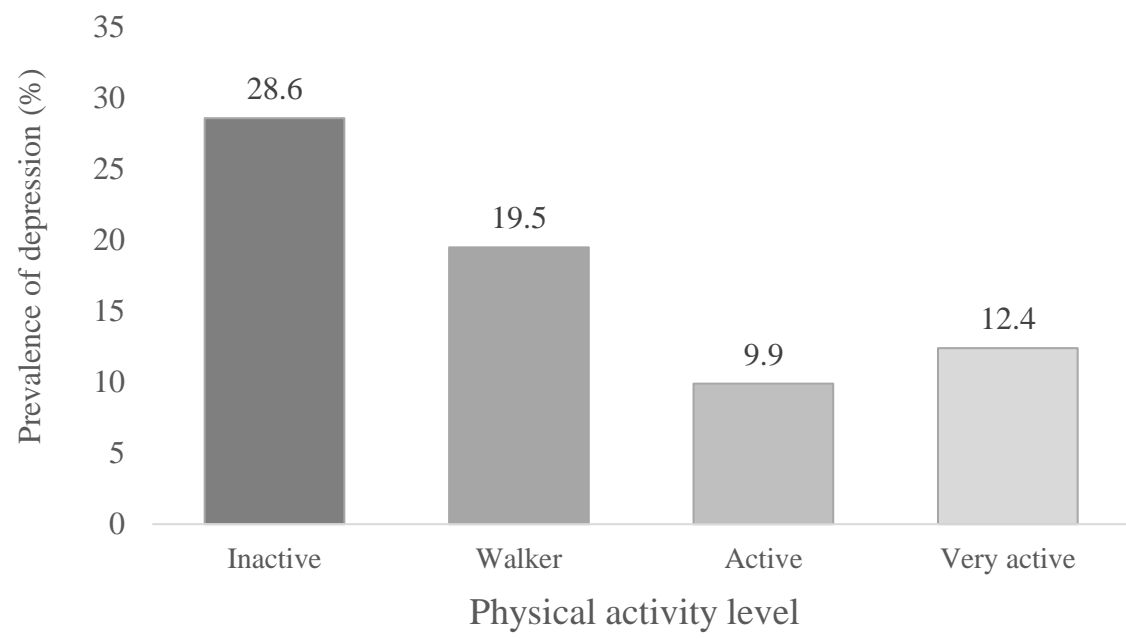

Supplement: Figure S1 [file peerj-12-17169-s002.pdf]

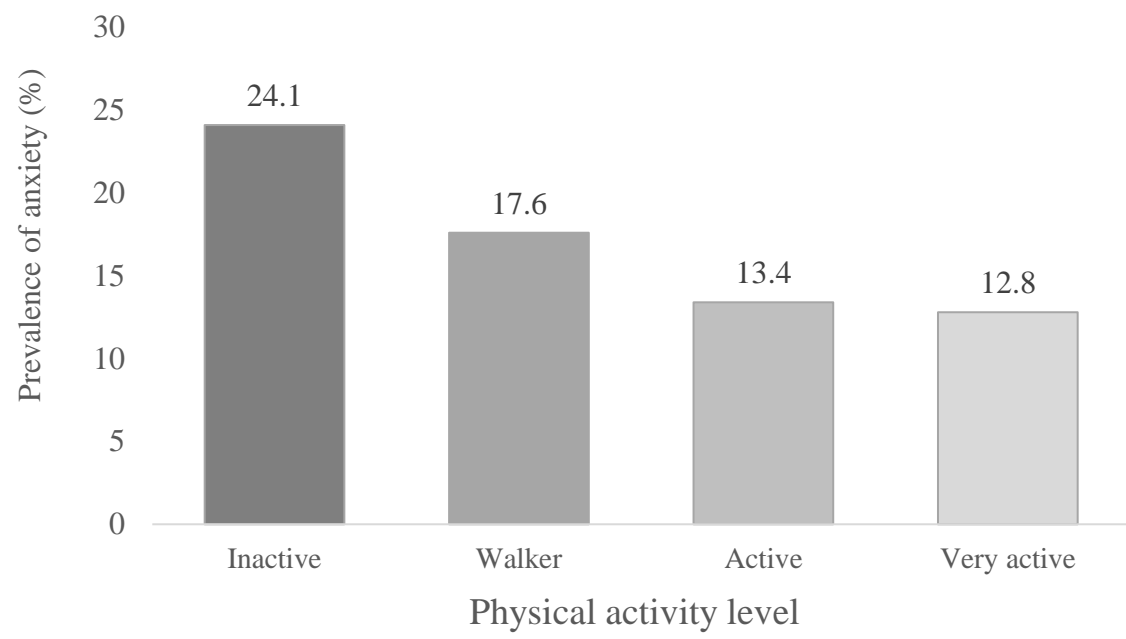

Supplement: Figure S2 [file peerj-12-17169-s003.pdf]

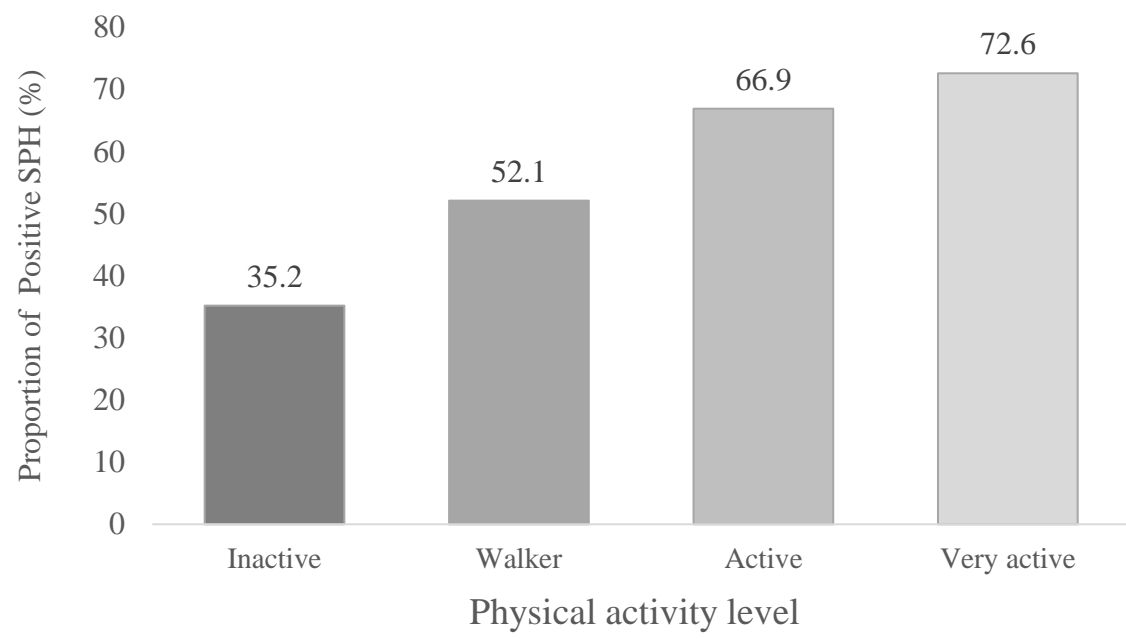

Supplement: Figure S3 [file peerj-12-17169-s004.pdf]
